# Evaluation of Clinical, Biochemical and Microbiological Markers Related to Dental Caries

**DOI:** 10.3390/ijerph18116049

**Published:** 2021-06-04

**Authors:** Maria D Ferrer, Salvadora Pérez, Aránzazu López Lopez, José Luis Sanz, Maria Melo, Carmen Llena, Alejandro Mira

**Affiliations:** 1 FISABIO Foundation, Center for Advanced Research in Public Health, 46020 Valencia, Spain; ferrer_mde@gva.es (M.D.F.); arantxazo2014@gmail.com (A.L.L.); mira_ale@gva.es (A.M.); 2 Department of Stomatology, Universitat de Valencia, 46010 Valencia, Spain; doraperez11@hotmail.es (S.P.); jsanzalex96@gmail.com (J.L.S.); llena@uv.es (C.L.)

**Keywords:** caries, *S. dentisani*, *S. mutans*, lactic acid, pH, dental plaque, saliva

## Abstract

Our aim was to evaluate clinical, biochemical and microbiological markers related to dental caries in adults. A sample that consisted of 75 volunteers was utilized. The presence of caries and the presence of plaque and gingival indices were determined. Unstimulated salivary flow, pH, lactate, *Streptococcus mutans* and *Streptococcus dentisani* were measured in the participants’ plaque and saliva samples before and after rinsing with a sugar solution. Lactate in plaque was found to be significantly related to age, gender, tooth-brushing frequency, the presence of cavitated caries lesions and plaque and gingival indices (*p* < 0.05). The levels of *S. dentisani* in plaque increased significantly with tooth-brushing frequency (*p* = 0.03). Normalized plaque *S. dentisani* values and the percentage of *S. dentisani* were slightly higher in patients with basal lactic acid levels ≤ 50 mg/L. After rinsing with a sugary solution, the percentage of *S. mutans* levels in plaque were higher in patients with lactic acid levels > 350 mg/L (*p* = 0.03). Tooth-brushing frequency was the factor which was most associated with oral health. Women reflected better clinical and biochemical parameters than men. Low pH and high lactic acid levels tended to be associated with high caries rates. No association was found between bacteria levels and caries indices.

## 1. Introduction

Dental caries is defined as a “biofilm-mediated, diet modulated, multifactorial, non-communicable, dynamic disease resulting in net mineral loss of dental hard tissues”. It is determined by biological, behavioral, psychosocial and environmental factors. As a consequence of this process, a caries lesion develops [1]. The transition from health to disease occurs when there is a disturbance that modifies the conditions of the oral environment and favors the development of a more acid-producing and acid-tolerant microbial community [2]. This potential for tolerance and production of acids cannot be attributed to a single group of microorganisms, but to a bacterial consortium that interacts in a complex manner and that, under certain conditions, would increase in proportion or activity, to the detriment of other bacteria whose metabolic output would be less acidogenic [3]. When the environmental conditions change, the oral microbiota can also change and reverse the effect.

In the biofilm of a caries lesion, *Streptococcus mutans* is not the most numerous species; there are many other microorganisms with aciduric and acidogenic potential that are also present [4]. However, *S. mutans* has been extensively studied as it is an important producer of extracellular matrix and can rapidly modulate the formation of a cariogenic biofilm when aided by the presence of fermentable carbohydrates in the diet. Sucrose is considered the most cariogenic sugar because, in addition to being fermented by oral bacteria, it is an excellent substrate for the synthesis of extracellular (EPS) and intracellular (IPS) polysaccharides [5,6]. In contrast, the presence of microorganisms with arginolytic and ureolytic activity in the biofilm can buffer the pH and present a high cario-preventive potential and this is due to the production of ammonia as a final metabolite [7]. The presence of sucrose, a low buffering capacity of saliva, and a low pH have been shown to be important factors that hinder the production of alkali from arginine [8]. Thus, the final cariogenic potential of an oral biofilm is not only determined by the presence of sugar-fermenting and acidogenic organisms but also by the levels of protective and pH-buffering bacteria in the oral cavity. However, the simultaneous quantification of bacterial biomarkers of both types of organisms is rarely performed.

The microbial diversity of a caries lesion is considerably lower than that of dental biofilms [9]. Within the complex microbiota that has been identified in genomic studies, it has been observed that a large part of the microorganisms involved are commensal. Many have not yet been cultivated and have not even been assigned to scientific nomenclature yet [10,11]. It has been possible to identify a series of microbial species in the metagenomic studies of dental plaque, which are more compatible with dental health than others [12]. In 2014 a new species of Streptococci from the mitis group was described and named as *Streptococcus dentisani* [13]. When this bacterium was isolated in pure culture and grown in the presence of cariogenic organisms, such as *S. mutans* or *Streptococcus sobrinus*, it was observed that the bacterium inhibited or killed pathogens by means of bacteriocins [14]. It has also been demonstrated that *S. dentisani* has the capacity to metabolize arginine into ammonia, which neutralizes dental plaque pH. Therefore, *S. dentisani* would provide a double anticariogenic mechanism; it would inhibit the growth of acidogenic bacteria, stimulate the formation of ammonium which results in a more favorable pH for dental health and it could act as a biomarker for beneficial oral bacteria [14].

Dental plaque constitutes the habitat in which microbial metabolic activity takes place and where both pathogenic and protective processes occur, which affects the development of the lesion. Urea, nitrate and arginine are the three main sources of alkali generation in plaque and saliva. Ammonia produced as a result of their metabolism can be an important endogenous inhibitory factor of the acidogenic microbiota and caries development by neutralizing acids and stabilizing the oral microbiota [15,16,17,18,19]. Thus, the levels of ammonia, pH and organic acids, such as lactate, could act as biomarkers to indicate the acidogenicity of oral communities and may be related to the risk of developing caries lesions. However, it is unclear whether the levels of these compounds have a better diagnostic and predictive value when measured in dental plaque or in saliva.

As early as 1940, Stephan reported that between 2 min and 15 min after rinsing with a sugary solution, the pH of dental plaque drops, lactic acid being mainly responsible for this drop, and then returns to its basal level around the 40 min mark [20]. This is possible thanks to the implementation of buffer mechanisms among which bicarbonate, urea and arginine stand out [18,21,22,23]. Thus, not only is the basal pH important as a measure of acidogenicity but also the level achieved after fermentation takes place. Accordingly, in the current manuscript pH and lactate levels before and after the pH drop caused by sugar exposure were measured.

The responsiveness of saliva and dental plaque when subjected to acid stress will be an indicator of the ability to compensate and balance acids in the oral environment either through the salivary compensatory mechanisms or the already described mechanisms of action of microorganisms such as *S. dentisani* [14]. To the authors’ knowledge, there are currently no studies that evaluate the possible association between the response to acid stress in saliva and plaque with the levels of *S. mutans* and *S. dentisani*, simultaneously, and its association with previous clinical records of caries and biochemical salivary and plaque parameters. Therefore, the present study aims to simultaneously analyze clinical, biochemical and microbiological factors potentially related to caries experience in a sample of adults. The following elements were assessed: plaque and gingival indices; salivary flow; salivary and plaque pH; and salivary and plaque lactate before and after sugar exposure. Additionally, the presence of *S. mutans* and the presence of *S. dentisani* in both plaque and saliva were quantified by quantitative PCR. Finally, the associations of all these parameters with dental caries experience, gender and tooth-brushing frequency were evaluated in order to better understand their relationships relative to one another and to identify potential biomarkers and risk predictors of the disease.

## 2. Materials and Methods

This study is part of two CECT 7746 clinical trials carried out in collaboration between the FISABIO Foundation and the Lluís Alcanyis Foundation of the Universitat de València. It was approved by the DGSP-CSISP Ethics Committee with project codes ABB-Sdent-Colonization and ABB-dentisani 2015. The study began in 2016 and was completed in 2018. Compliance of the protocol and surveillance of the study was performed by the external CRO Effice (Madrid, Spain).

### 2.1. Participant Selection Process

In order to carry out the present study, 184 volunteers were recruited. Informed consent was obtained from all subjects involved in the study. Inclusion criteria for the selection of participants were: age between 18 and 65 years; basal salivary pH (after brushing with water) ≤ 7; at least 21 teeth present in the oral cavity; previous caries experience and/or presence of active caries lesions; and the absence of other oral diseases. Patients with chronic diseases; use of medication or previous procedures, such as head and neck radiotherapy or pathologies that reduce salivary flow; basal pH at the time of recruitment > 7; and/or absence of previous caries experience; and/or active caries lesions were excluded. Individuals taking antibiotics during the previous 3 months or regular use of oral antiseptic mouthwashes during the previous week were also excluded. After applying the inclusion and exclusion criteria, a total of 75 participants were selected.

### 2.2. Clinical Examination and Sample Collection

After being included in the study, each participant received an appointment to which he/she had to attend without having brushed his/her teeth since the night before. In this appointment, which was carried out between 4:00 p.m. and 7:00 p.m., frequency of daily tooth-brushing was recoded. A sample of unstimulated basal saliva obtained by drooling was taken for five minutes to determine salivary flow by considering values from 0.25 to 0.3 mL/min as the normal secretion rates (sample 0). From these salivary samples, the buffer pH was determined.

Subsequently, an oral examination was performed and the plaque and gingival index of Silness and Löe and Löe and Silness [24], respectively, were determined. Additionally, the supragingival biofilm of all teeth surfaces was collected in two 1.5 mL Eppendorf tubes using a sterile dental excavator. Biofilm from quadrants 1 and 3 were deposited in 100 µL phosphate buffer solution (PBS) for its conservation until the subsequent extraction of bacterial DNA. Biofilm from quadrants 2 and 4 were deposited in 100 µL sterile H_2_O (pH = 7) for the subsequent determination of pH and lactic acid content after sugar exposure.

After the plaque sample collection, patients were instructed to brush their teeth 2 min with water using a manual toothbrush. The presence of caries was then assessed using the ICDAS II criteria by the same experienced explorer in all participants [25].

Next, an additional sample of unstimulated saliva obtained by drooling (sample 1) was collected, in which the pH was determined. The participants then rinsed with a 10% sugar solution for 1 min and 10 min after rinsing a final salivary sample was collected (sample 2).

### 2.3. Determination of Salivary pH

Salivary pH was determined from samples 0, 1 and 2 by means of a reflectometer (Reflectoquant; Merck, Darmstadt, Germany) which was calibrated with the corresponding pH strips (reference: 116996).

### 2.4. Determination of Biofilm pH and Lactate Content

For the determination of biofilm lactate content, 30 µL of the initial sample (100 µL H_2_O + biofilm) was deposited onto the test strips. After 7 min, they were introduced into the reflectometer for their measurement (t0) after calibration with the lactic acid strips (reference: 116127). For the measurement of biofilm pH, 30 µL of the same sample was deposited onto the corresponding test strips and, after 10 s, they were introduced into the reflectometer. Then, 40 µL of 20% sucrose solution was added to the initial sample, thus obtaining a final sucrose concentration of 10%. Immediately afterwards, the samples were introduced in a laboratory oven at 37 °C for a 10 min incubation period. After the incubation period, the pH and lactic acid (t10) were measured again.

### 2.5. Count of S. mutans, S. dentisani, and Total Bacterial Count

The microorganism count was performed in plaque and saliva samples: 250 µL of basal saliva (sample 0) and the plaque samples from quadrants 1/3 resuspended in 100 µL of PBS buffer were used. First, the DNA was extracted in an automated process, using the MagnaPure LC JE379 equipment and the MagnaPure LC DNA Isolation Kit that are both from Roche (Basel, Switzerland), after an enzymatic lysis [26]. A fluorometric method (Quant-iT PicoGreen dsDNA Assay, Invitrogen) was used to quantify the extracted DNA. The reactions for the quantification of *S. mutans*, *S. dentisani* and total bacteria were carried out by means of qPCR (quantitative Polymerase Chain Reaction) with the LightCycler 480 equipment and the LightCycler 480 SYBR Green I Master Mix kit (Roche, Basel, Switzerland).

The specific primers used for the quantification of *S. dentisani* (CkSdF and CkSdR) [14] amplify a 77 base pair region of the carbamate kinase gene. For the quantification of *S. mutans*, primers which were already reported in the literature were used which amplify a 415 base pair fragment of the glycosyl transferase gene [27]. In the case of total bacteria, the target gene was the 16S rDNA ribosomal gene that is highly conserved in the Bacteria Domain. The primers used (515F-789R) amplify a region of 274 base pairs [28,29].

All amplification reactions were carried out in a final volume of 20 µL containing 1 µL of template DNA (5–22 ng/µL), 10 µL of LightCycler 480 SYBR Green I Master Mix, 0.4 µL of each primer and 7.2 µL of nuclease-free water. The thermocycling program used is described as follows: an initial denaturation step at 95 °C for 5 min, 40 cycles of 10 s at 95 °C, 20 s at 65 °C (for total bacteria it was reduced to 58 °C) and 25 s at 72 °C. All reactions were performed in duplicates as well as their corresponding positive and negative controls.

### 2.6. Statistical Analysis

The ICDAS II values were recalculated as Decayed, Missing and Filled Teeth (DMFT) values. In component D, cavitated caries (caries ICDAS codes 3–6 and restauration ICDAS codes 7 and 8) on the one hand and cavitated and not cavitated caries on the other (caries ICDAS codes 1–6 and restauration ICDAS codes 7 and 8) were recoded. In components M and F, ICDAS code 97 and restoration ICDAS codes 3–6, respectively, were included [30].

Using the Kolmogorov–Smirnov test, it was determined if the quantitative variables did not follow a normal distribution. Non-parametric tests were used if this was the case. The Kruskal–Wallis test was used to compare tooth-brushing frequency and quantitative variables. The Mann–Whitney U test was used to compare gender with the quantitative variables. Chi-squared test was used for comparison between gender and tooth-brushing frequency. Box plot graphs representing median and Inter quartile range (IQR) were used for presenting data. Finally, correlation analyses were performed between the different study parameters using the Spearman correlation coefficient. In all cases, a significance level of 95% was used.

## 3. Results

The sample consisted of 24 men (32%) and 51 women (68%) with a mean age of 34.72 ± 10.84 years.

### 3.1. Analysis of Clinical, Biochemical and Microbiological Variables by Gender

**Figure 1:**
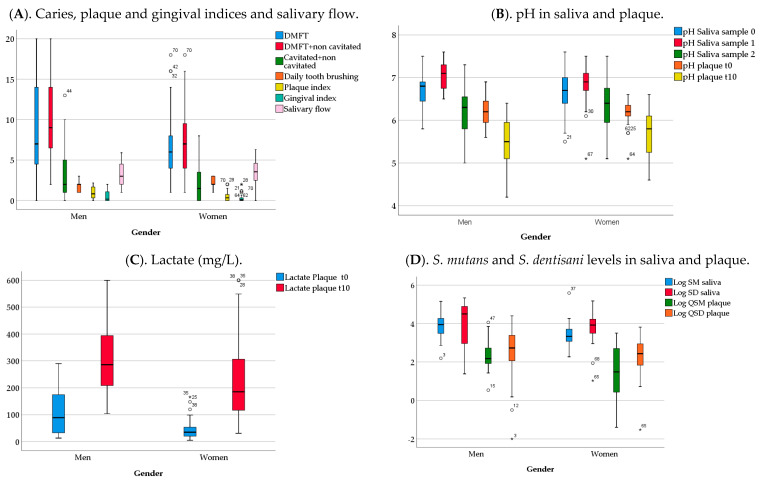
Clinical, biochemical and microbiological variables by gender. The graphs show medians and IQR values for the different variables. (**A**) DMFT = ICDAS codes 3–6 + restoration ICDAS codes 7 and 8 + missing teeth due to caries + filled teeth due to caries. DMFT with non-cavitated lesions = DMFT + ICDAS codes 1–6 + restoration ICDAS codes 7 and 8. (**B**) pH_saliva (sample 0) = basal salivary pH. pH_saliva (sample 1) = salivary pH after tooth-brushing. pH_saliva (sample 2) = salivary pH after sugar rinse. pH plaque t0 = plaque pH before sugar rinse. pH plaque t10 = plaque pH after sugar rinse. (**C**) Lactate plaque t0 = plaque lactate levels before sugar rinse (mg/L). Lactate plaque t10 = plaque lactate levels after sugar rinse (mg/L). (**D**) Log SM saliva = Log10 transformation of normalized CFUs/mL of *S. mutans* in saliva, Log SD saliva = Log10 transformation of normalized CFUs/mL of *S. dentisani* in saliva. Log QSM plaque = Log10 transformation of normalized CFUs/ng of *S. mutans* in plaque, Log QSD plaque = Log10 transformation of normalized CFUs/ng of *S. dentisani* in plaque. The expected loss in statistical power due to the difference in male:female ratio is <0.05.

Analyses by gender revealed that caries indices and their components were higher for men than for women, except for the filled teeth that were slightly higher for women. After analyzing their components, missing teeth values for men were significantly higher than for women (*p* = 0.01). Plaque index was significantly higher in men than in women (*p* = 0.01). Men brushed their teeth significantly less frequently than women (*p* = 0.02, Chi-squared test) (Figure 1A). The pH of saliva or of plaque at different time points were similar in men and in women (Figure 1B). Plaque lactate levels at both t0 and t10 were significantly higher for men (Mann–Whitney U test) (*p* < 0.01) (Figure 1C). The levels of *S. mutans* and *S. dentisani* were obtained in saliva normalizing by volume (CFUs/mL) and in plaque after normalizing by the total bacterial DNA present (CFUs/ng). Values for *S. mutans* were significantly higher in men than in women (*p* < 0.01). Figure 1D shows median and IQR for the levels of *S. mutans* and *S. dentisani* transformed in log10 values.

### 3.2. Analysis of Clinical, Biochemical and Microbiological Variables by Tooth-Brushing Frequency

**Figure 2:**
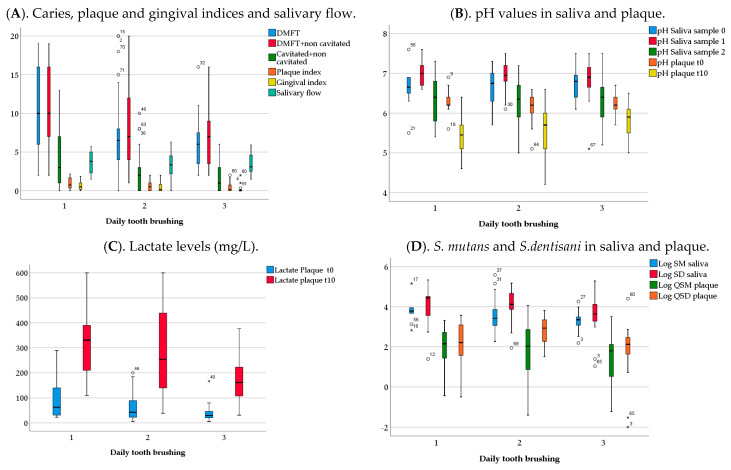
Clinical, biochemical and microbiological variables by daily tooth-brushing. Graphs show medians and IQR values for the different variables. (**A**) DMFT = ICDAS codes 3–6 + restoration ICDAS codes 7 and 8 + missing teeth due to caries + filled teeth due to caries. DMFT with non-cavitated lesions = DMFT + ICDAS codes 1–6 + restoration ICDAS codes 7 and 8. (**B**) pH_saliva (sample 0) = basal salivary pH. pH_saliva (sample 1) = salivary pH after tooth-brushing. pH_saliva (sample 2) = salivary pH after sugar rinse. pH plaque t0 = plaque pH before sugar rinse. pH plaque t10 = plaque pH after sugar rinse. (**C**) Lactate plaque t0 = plaque lactate levels before sugar rinse (mg/L). Lactate plaque t10 = plaque lactate levels after sugar rinse (mg/L). (**D**) Log SM saliva = Log10 transformation of normalized CFUs/mL of *S. mutans* in saliva, Log SD saliva= Log10 transformation of normalized CFUs/mL of *S. dentisani* in saliva. Log QSM plaque = Log10 transformation of normalized CFUs/ng of *S. mutans* in plaque, Log QSD plaque = Log10 transformation of normalized CFUs/ng of *S. dentisani* in plaque.

Participants who brushed their teeth two or three times a day presented lower values of DMFT (with or without non cavitated lesions) than those that brushed only once a day. Components D and M were also lower in these patients (*p* < 0.05). Plaque and gingival indices were significantly lower in participants who brushed their teeth three times a day compared to those that brushed once per day (*p* < 0.05) (Figure 2A). Lactate values at t10 decreased significantly when the tooth-brushing frequency increased (*p* < 0.05) (Figure 2C). The pH values and normalized levels of *S. mutans* and *S. dentisani* in saliva (CFUs/mL) or in plaque (CFUs/ng) did not vary significantly with tooth-brushing frequency (Figure 2B,D).

### 3.3. Analysis of Clinical and Biochemical Variables

Table 1 shows the correlation analysis of the different clinical and biochemical variables analyzed (Spearman’s correlation coefficient). A positive significant correlation was found between plaque index and gingival index and plaque pH and lactate levels at t0 and t10 (*p* < 0.05). The basal saliva pH (sample 0) was significantly positive correlated with saliva pH after brushing (sample 1) and after sugar rinse (sample 2), respectively (*p* < 0.05). Plaque pH and lactate levels at t0 and t10 were also significantly positive correlated (*p* < 0.05).

### 3.4. Microbiological Parameters

The levels of *S. mutans* and *S. dentisani* in saliva (CFUs/mL) and plaque (CFUs/ng) were determined after normalizing the quantification according to the total bacterial DNA present. The percentage of both microorganisms was also calculated according to the total number of bacteria present in the plaque material. The levels of *S. dentisani* in plaque increased significantly when tooth-brushing frequency increased (7.71 × 10^2^/tooth-brushing once a day, 1.42 × 10^3^/tooth-brushing twice or three times a day) (*p* = 0.03) (the same trends were observed in the percentages of *S. dentisani* in plaque) (0.29 %/tooth-brushing once a day, 0.42 %/tooth-brushing two or three times a day) (*p* = 0.04) (Kruskal–Wallis test). Values of *S. dentisani* (1.38 × 10^3^/1.07 × 10^3^) and percentages of *S. dentisani* in plaque (0.42%/0.32%) were higher in patients with DMFT levels ≤ 8. Levels of *S. mutans* in saliva were higher in patients with baseline salivary pH below 6.4 (5.29 × 10^3^/1.57 × 10^4^). Plaque normalized *S. dentisani* values and the percentages of *S. dentisani* were slightly higher in patients with lactic acid levels ≤ 50 mg/L. After the sugar rinse, percentages of *S. mutans* levels in plaque were higher in patients with lactic acid levels > 350 mg/L (*p* = 0.03) (Mann–Whitney U test).

Table 2 shows the correlations between microbiological parameters and clinical and biochemical parameters (Spearman correlation analysis). Age was found to be significantly correlated with *S. dentisani* levels in saliva and plaque. Plaque index was observed to be significantly related to *S. mutans* and *S. dentisani* levels in saliva and the percentages of *S. dentisani* in plaque. The levels of *S. mutans* and *S. dentisani* in saliva were significantly associated, as were *S. mutans* levels in plaque and their percentages. Basal pH in saliva (sample 0) was significantly and inversely associated with *S. dentisani* levels. The pH in plaque at t0 was found to be significantly and inversely associated with *S. dentisani* levels in saliva and plaque and with *S. mutans* in plaque. pH at t10 was found to be significantly and inversely associated with microbiological levels in plaque and saliva. Lactate values at t0 and t10 were significantly correlated with microbiological levels in saliva and *S. mutans* levels in plaque.

## 4. Discussion

The mean age of the study sample corresponds to the group of young adults proposed by the WHO, which includes the population between 35 and 44 years old. If that population group is taken as a reference in the data obtained in the last national survey carried out in 2020 then the average DMFT index was 7.40 ± 4.86, which is very similar to our sample where the mean DMFT values were 7.45 ± 4.83. The mean values of cavitated and filled lesions were similar to those from the national study for the young adult population, while the mean values of missing teeth were lower: 0.73 vs. 1.92 [31]. In the study of Eustaquio M.V. et al. in 2010 on the oral health of the population of the Valencian Region (Spain), similar DMFT values were observed in young adults [32] to those of the present study (7.45 vs. 7.64).

With regards to the association between gender and tooth-brushing frequency, it was observed that men brushed less frequently than women. It was also observed that men had higher DMFT values and its components, with the exception of the restoration index which was higher among women. However, a significant difference was only found in component M (missing to caries). These data are consistent with those found in the 2020 national epidemiological study [31]. In different studies, the direct relationship between tooth-brushing habits and the previous experience of caries has been verified [33,34,35,36]. Regarding the plaque and gingival indices, these were also higher in men although the difference was only significant for the plaque index. These data are consistent with those found in the literature [31,36,37]. However, a limitation of the present study could be not having taken into account other relevant factors in caries etiology such as diet, social and behavioral factors as well as the difference in sample size between men and women. Thus, although the microbiological and biochemical features measured in the current study correspond only to 75 individuals, this agreement in clinical data with the national survey suggests that we are working on a representative set of individuals.

Additionally, significantly higher levels of *S. mutans* were found in plaque and in saliva in men than compared to women. These data are correlated with the results obtained regarding the plaque index. Good oral hygiene habits control the oral microbiota at levels compatible with health [38] and our data suggest better oral hygiene parameters in women, which is translated in better clinical and microbiological features. Likewise, lactate levels were higher in men both before and after the sugar rinse. This can be explained because higher concentrations of lactate are related to a greater amount of plaque with acidogenic properties. It is well established that acidogenic and aciduric biofilms are associated with an increased risk of disease [39]. In a recent study that microbiologically evaluated 268 young patients in the Netherlands, a strong gender pattern was also observed in microbial composition and metabolism and more cariogenic microbiota was observed in men resulting in significant pH differences [40].

With the data obtained in the present study, it can be highlighted that factors associated with gender influences the incidence of dental caries; it is observed that men have poorer oral health and that women take greater care of their oral hygiene both individually (tooth-brushing) and professionally (restorations). Nevertheless, other factors such as dietary habits or sociocultural and behavioral factors should also be considered when estimating the risk of developing caries.

After analyzing the tooth-brushing frequency with the quantitative variables, it was observed that the participants who brushed their teeth once a day had worse oral health than those who did so 2–3 times a day. In addition, participants who brushed their teeth three times a day had a significantly lower gingival index and lower plaque lactate content. The beneficial effect of tooth-brushing on the risk of caries has been demonstrated in numerous studies and is the most effective method for removing plaque [41] and the best fluoride delivery system [42]. Consequently, it can be concluded that brushing three times a day reduces the risk of tooth decay and that brushing frequency is predictive of caries risk. In agreement with this, it is interesting that the levels of the cariogenic bacterium *S. mutans* corelated negatively with tooth-brushing frequency but positively with the health-associated species *S. dentisani*.

When correlating the clinical and laboratory variables, it was observed that DMFT increases with age, that tooth absences are significantly related to plaque and gingival indices and that plaque and gingival indices have a significant association with pH levels and lactate content before and after the sugar rinse. In 1987, Firestone et al. verified how the maturity of dental plaque considerably increases the drop in pH after a meal ingestion [43]. This fact supports the correlation observed between plaque index, pH and lactate content of the plaque itself. Experimental studies have also demonstrated the association between certain microbial components of mature plaque and the ability to modify pH, lactic acid production and the presence of enzymes with high pathogenic potential [3].

Among the factors associated with microbiology, lactate is the most reliable marker of caries. Given that this marker was measured in plaque and that *S. mutans* levels were better correlated with caries parameters when this bacterium was quantified in plaque, the present work supports the use of dental plaque to measure microbiological parameters with greater reliability than saliva, which showed erratic and inconsistent trends in the different factors measured. Despite this fact, it is interesting that the levels of both *S. mutans* and *S. dentisani* in plaque were significantly correlated with the values of these bacteria in saliva. This is possibly due to the fact that both species are mainly inhabitants of hard tissues and, therefore, their higher levels in plaque imply a greater release in saliva. Other streptococci which are found at high levels in soft tissues, such *S salivarius*, may not correlate between these two niches.

Microbiological data support that, despite the fact that *S. mutans* is a minor inhabitant of dental plaque representing less than 1% of the total microbiota, its presence is correlated with lactate production and is therefore an important bacterium in acid generation [12]. However, the correlations of this bacterium with caries levels were neither positive nor significant, neither with pH values nor with caries levels or caries indices. Future studies should include the levels of other acidogenic bacteria in order to establish the microbiological risk of caries more accurately. It was observed, for example, that the measurement of *S. mutans* and *Lactobacillus* in a combined manner correlates more with caries parameters than each one separately [44]. Other authors are trying to incorporate other bacterial species in the map of the prediction of the risk of suffering from the disease [45].

The levels of *S. dentisani* were higher in individuals without restorations, which could indicate a negative association with the history of caries, while the opposite pattern was detected in *S. mutans*. However, these data were inconsistent with the levels of active caries or cavitated caries and thus there is no clear protective relationship between the presence of *S. dentisani* and caries in individuals who already have the disease. Given that there are higher levels of *S. dentisani* in individuals without caries [13], it is possible that the beneficial effect of *S. dentisani* requires minimal levels of the bacteria to be effective although it cannot be ruled out that said protective effect depends on the strain of *S. dentisani* present in each individual.

Unlike what occurred with *S. mutans*, *S. dentisani* levels did not correlate with lactate production in plaque, although the percentage of *S. dentisani* in plaque was higher in patients with lower lactate levels both before and after the sugar rinse. However, the levels of both bacteria before and after the sugar rinse were negatively related to plaque pH. This suggests that pH is related to the total amount of plaque or bacterial load, regardless of whether or not the protective effect of *S. dentisani* was present.

In in vitro experiments, it has been shown that the buffer effect of *S. dentisani* through the arginine pathway requires several hours [14] and therefore would not be appreciated in the data of the present work. A second explanation for this lack of correlation between pH and levels of *S. dentisani* would be given by the absence of individuals without caries [DMFT = 0] in the study since it is these individuals who have shown higher levels of *S. dentisani* and of arginolytic activity in general [18,46]. Therefore, the data would support the idea that once the disease is present, the levels of *S. dentisani* in these individuals are not sufficient to prevent either *S. mutans* levels or lactate production or to maintain a more alkaline basal pH. In fact, it is surprising that despite the substantial inhibition produced by this bacterium against cariogenic organisms under laboratory conditions, there is no negative correlation between the presence of *S. dentisani* and the levels of *S. mutans*. Future work should therefore establish this correlation in individuals who have never suffered caries.

In a recently published article, it has been shown that the administration of a bioadhesive gel, applied with individualized trays for a week, containing the probiotic *S dentisani* causes an increase in salivary pH, a reduction in the production of lactic acid up to 30 days after its application, a reduction in the colonization of *S mutans* and an increase in the presence of *S dentisani* up to 14 days after its application [47]. In a second clinical trial where the probiotic was applied for a month, improvements in plaque and gingival indices, as well as in salivary ammonia and pathogen levels were observed [48] and this suggests, again, that there is minimum level of this organism that is necessary to observe an improvement in clinical and microbiological features.

Meanwhile, in patients who already have the disease, our data strongly confirm that tooth-brushing is the most effective method to improve caries risk and the development of cavities [41,42]. In this sense, the significant correlation that exists between tooth-brushing and *S. dentisani* levels is interesting and it indicates that this bacterium is favored by correct oral hygiene. This suggests that it is an early colonizer of the plaque and/or that it is favored by the lower frequency of a mature and acidogenic plaque. Therefore, a synergistic effect could occur between tooth-brushing and the presence of *S. dentisani* as it has been observed after tongue-brushing for other beneficial organisms such as *Rothia* [48]. Given that *S. dentisani* uses arginine for its pH buffering action [45], it would be interesting to test whether the use of arginine toothpastes and the administration of *S. dentisani* as a probiotic can have a synergistic effect in individuals with cavities.

## 5. Conclusions

Tooth-brushing frequency was the most decisive factor associated with oral health. Low pH and high lactic acid levels tended to be associated with higher caries rates. However, no association was found between the levels of specific bacteria and dental caries and this suggests that a single bacterial species cannot be used as a valid biomarker of the disease.

## Figures and Tables

**Table 1:** Analysis of the correlations (Correlation Coefficients (CC) and *p*-values) between the quantitative variables assessed. Significant correlations are bolded.

		Age	D_Cavitated ^1^	D_non Cavitated ^2^	M ^3^	F ^4^	DMFT ^5^	DMFT + Non Cavitated ^6^	PI ^7^	GI ^8^	Salivary Flow	pH_Saliva (Sample 0) ^9^	pH_Saliva (Sample 1) ^10^	pH_Saliva (Sample 2) ^11^	pH_Plaque_t0 ^12^	pH_Plaque_t10 ^13^	Lactate_ Plaque_t0 ^14^
**D_cavitated**	CC	0.11															
	p	0.34															
**D_non cavitated**	CC	−0.17	−0.20														
	p	0.13	0.07														
**M**	CC	**0.60**	0.08	−0.02													
	p	**0.00**	0.48	0.80													
**F**	CC	0.17	**−0.32**	−0.14	0.20												
	p	0.14	**0.00**	0.23	0.08												
**DMFT**	CC	**0.33**	**0.262**	**−0.23**	**0.50**	**0.69**											
	p	**0.00**	**0.02**	**0.04**	**0.00**	**0.00**											
**DMFT + non cavitated**	CC	**0.27**	0.19	0.15	**0.54**	**0.61**	**0.887**										
	p	**0.01**	0.10	0.19	**0.00**	**0.00**	**0.00**										
**D cavitated + non cavitated**	CC	−0.03	**0.72**	**0.42**	0.06	**−0.32**	0.15	**0.36**									
	p	0.79	**0.00**	**0.00**	0.59	**0.00**	0.17	**0.00**									
**PI**	CC	0.21	**0.39**	−0.13	**0.26**	−0.09	**0.23**	0.20									
	p	0.06	**0.00**	0.26	**0.02**	0.39	**0.03**	0.07									
**GI**	CC	0.18	0.16	−0.13	**0.24**	−0.07	0.17	0.14	**0.53**								
	P	0.11	0.16	0.24	**0.03**	0.51	0.14	0.23	**0.00**								
**Salivary flow**	CC	−0.03	0.08	0.08	0.09	0.06	0.16	0.15	−0.15	0.07							
	p	0.77	0.46	0.46	0.42	0.58	0.14	0.18	0.19	0.51							
**pH_saliva (sample 0)**	CC	−0.15	−0.12	0.07	0.01	−0.06	−0.09	−0.10	−0.10	0.05	0.07						
	p	0.19	0.27	0.53	0.93	0.58	0.41	0.39	0.36	0.66	0.52						
**pH_saliva (sample 1)**	CC	−0.08	−0.05	0.15	0.05	−0.20	−0.12	−0.07	0.00	0.02	0.03	**0.56**					
	p	0.47	0.63	0.17	0.65	0.08	0.28	0.53	0.98	0.84	0.75	**0.00**					
**pH_saliva (sample 2)**	CC	−0.25	0.05	0.15	−0.15	−0.21	−0.21	−0.17	−0.09	0.00	0.18	**0.32**	**0.39**				
	p	0.02	0.66	0.19	0.17	0.06	0.06	0.13	0.41	0.94	0.10	**0.00**	**0.00**				
**pH_plaque_t0**	CC	−0.03	−0.03	0.04	0.06	0.15	0.08	0.11	**−0.24**	−0.07	0.03	−0.02	0.00	−0.04			
	p	0.79	0.79	0.68	0.56	0.18	0.48	0.32	**0.03**	0.54	0.78	0.81	0.99	0.72			
**pH_plaque_t10**	CC	−0.18	−0.14	0.05	−0.22	0.04	−0.10	−0.10	**−0.39**	**−0.27**	0.12	−0.08	**−0.26**	−0.03	**0.53**		
	p	0.11	0.21	0.61	0.05	0.67	0.38	0.37	**0.00**	**0.01**	0.28	0.44	**0.02**	0.79	**0.00**		
**Lactate_plaque_t0**	CC	**0.31**	0.17	−0.16	**0.27**	0.03	0.16	0.16	**0.44**	**0.35**	−0.17	−0.05	0.01	−0.10	−0.018	**−0.59**	
	p	**0.00**	0.13	0.16	**0.01**	0.75	0.16	0.16	**0.00**	**0.00**	0.13	0.65	0.87	0.39	0.11	**0.00**	
**Lactate_plaque_t10 ^15^**	CC	**0.29**	**0.28**	−0.11	**0.29**	−0.07	0.17	0.15	**0.39**	**0.34**	−0.03	0.02	0.17	0.01	−0.19	**−0.67**	**0.75**
	p	**0.01**	**0.01**	0.33	**0.00**	0.53	0.13	0.17	**0.00**	**0.00**	0.77	0.83	0.14	0.88	0.10	**0.00**	**0.00**

^1^ D_cavitated = cavitated caries. ^2^ D non cavitated = non-cavitated caries. ^3^ M = missing teeth due to caries. ^4^ F = filled teeth due to caries. ^5^ DMFT = ICDAS codes 3–6 + missing due to caries + filled due to caries teeth. ^6^ DMFT + non cavitated lesions = DMFT + ICDAS scores 1–2. ^7^ PI = plaque index. ^8^ GI = gingival index. ^9^ pH_saliva (sample 0) = basal salivary pH. ^10^ pH_saliva (sample 1) = salivary pH after tooth-brushing. ^11^ pH_saliva (sample 2) = salivary pH after sugar rinse. ^12^ pH_plaque_t0 = plaque pH before sugar rinse. ^13^ pH_plaque_t10 = plaque pH after sugar rinse. ^14^ Lactate_plaque_t0 = plaque lactate levels before sugar rinse. ^15^ Lactate_plaque_t10 = plaque lactate levels after sugar rinse. CC: correlation coeficient. Values in bold are statistically significant (*p* < 0.05).

**Table 2:** Correlation analysis (Correlation Coefficient, CC and *p*-value) between the levels of *S. mutans* and *S. dentisani* in saliva or plaque with different caries indices and biochemical parameters.

		*S. mutans* Saliva	*S. dentisani* Saliva	*S. dentisani* norm. in Plaque	*S. mutans* norm. in Plaque	% *S. dentisani* in Plaque	% *S. Mutans* in Plaque
**Age**	CC	0.154	0.311	0.220	−0.105	0.254	−0.084
	p	0.200	**0.008**	0.065	0.385	**0.033**	0.486
**PI ^1^**	CC	0.27	0.412	0.226	0.098	0.246	0.040
	p	**0.020**	**0.000**	0.058	0.415	**0.039**	0.742
**GI ^2^**	CC	0.168	0.108	0.088	0.046	0.098	0.000
	p	0.162	0.370	0.467	0.706	0.415	0.998
**Salivary flow**	CC	−0.034	−0.197	−0.036	0.048	−0.142	−0.015
	p	0.777	0.100	0.763	0.688	0.236	0.899
**D_cavitated ^3^**	CC	0.242	0.349	0.107	−0.046	0.159	−0.045
	p	**0.042**	**0.003**	0.373	0.705	0.185	0.709
**D_cavitaed + non cavitated ^4^**	CC	0.210	0.260	0.186	0.068	0.245	0.027
	p	0.079		0.119	0.574	**0.039**	0.823
**M ^5^**	CC	0.149	0.150	0.068	0.085	0.018	0.114
	p	0.214	0.211	0.574	0.479	0.882	0.346
**F ^6^**	CC	−0.123	−0.234	−0.120	0.003	−0.128	0.040
	p	0.308	**0.049**	0.320	0.981	0.287	0.738
**DMFT ^7^**	CC	0.084	0.044	−0.049	0.007	−0.074	0.058
	p	0.488	0.718	0.688	0.951	0.539	0.633
**DMFT_with non cavitated ^8^**	CC	0.110	0.037	−0.001	0.053	−0.012	0.084
	p	0.363	0.760	0.993	0.659	0.919	0.484
***S. mutans* in saliva**	CC		0.594	0.116	0.488	0.013	0.460
	p		**0.000**	0.335	**0.000**	0.913	**0.000**
***S. dentisani* in saliva**	CC	0.594		0.399	0.181	0.429	0.179
	p	**0.000**		**0.001**	0.131	**0.000**	0.136
***S. dentisani*** **norm. in plaque**	CC	0.116	0.399		0.300	0.893	0.160
	p	0.335	**0.001**		**0.011**	**0.000**	0.182
***S. mutans*** **norm. in plaque**	CC	0.488	0.181	0.300		0.151	0.922
	p	**0.000**	0.131	**0.011**		0.208	**0.000**
**% *S. dentisani* in plaque**	CC	0.013	0.429	0.893	0.151		0.079
	p	0.913	**0.000**	**0.000**	0.208		0.512
**% *S. mutans* in plaque**	CC	0.460	0.179	0.160	0.922	0.079	
	p	**0.000**	0.136	0.182	**0.000**	0.512	
**pH_saliva (sample 0) ^9^**	CC	−0.177	−0.238	−0.103	−0.111	−0.124	−0.154
	p	0.139	**0.046**	0.392	0.359	0.302	0.201
**pH_saliva (sample 1) ^10^**	CC	0.146	−0.131	−0.044	0.132	−0.113	0.125
	p	0.223	0.278	0.715	0.273	0.348	0.298
**pH_saliva (sample 2) ^11^**	CC	−0.167	−0.216	−0.020	−0.111	−0.018	−0.172
	p	0.165	0.071	0.867	0.357	0.879	0.152
**pH_plaque_t0 ^12^**	CC	−0.207	−0.351	−0.411	−0.289	−0.296	−0.230
	p	0.084	**0.003**	**0.000**	**0.015**	**0.012**	0.053
**pH_plaque_t10 ^13^**	CC	−0.368	−0.286	−0.286	−0.427	−0.177	−0.394
	p	**0.002**	**0.016**	**0.016**	**0.000**	0.141	**0.001**
**Lactate_plaque t0 ^14^**	CC	0.333	0.324	0.045	0.249	0.112	0.332
	p	**0.005**	**0.006**	0.710	**0.036**	0.353	**0.005**
**Lactate_plaque t10 ^15^**	CC	0.362	0.402	0.137	0.246	0.148	0.293
	p	**0.002**	**0.001**	0.254	**0.039**	0.218	**0.012**

^1^ PI= plaque index. ^2^ GI = gingival index. ^3^ D_cavitated = cavitated caries. ^4^ D_non cavitated = non-cavitated caries. ^5^ M = missing teeth due to caries. ^6^ F = filled teeth due to caries. ^7^ DMFT = ICDAS scores 3–6 + missing due to caries + filled due to caries teeth. ^8^ DMFT + non cavitated lesions = DMFT + ICDAS scores 1–2. ^9^ pH_saliva (sample 0) = basal salivary pH. ^10^ pH_saliva (sample 1) = salivary pH after tooth-brushing. ^11^ pH_saliva (sample 2) = salivary pH after sugar rinse. ^12^ pH_plaque_t0 = plaque pH before sugar rinse. ^13^ pH_plaque_t10 = plaque pH after sugar rinse. ^14^ Lactate_plaque_t0 = plaque lactate levels before sugar rinse. ^15^ Lactate_plaque_t10 = plaque lactate levels after sugar rinse, CC: correlation coefficient. Values in bold are statistically significant (*p* < 0.05).

## Data Availability

The data presented in this study are available upon request from the corresponding author.
